# Cost Effectiveness of Implementing Integrated Management of Neonatal and Childhood Illnesses Program in District Faridabad, India

**DOI:** 10.1371/journal.pone.0145043

**Published:** 2016-01-04

**Authors:** Shankar Prinja, Pankaj Bahuguna, Pavitra Mohan, Sarmila Mazumder, Sunita Taneja, Nita Bhandari, Henri van den Hombergh, Rajesh Kumar

**Affiliations:** 1 School of Public Health, Post Graduate Institute of Medical Education and Research (PGIMER), Chandigarh, India; 2 UNICEF India Country Office, New Delhi, India; 3 Centre for Health Research and Development, Society for Applied Studies, New Delhi, India; Centre Hospitalier Universitaire Vaudois, FRANCE

## Abstract

**Introduction:**

Despite the evidence for preventing childhood morbidity and mortality, financial resources are cited as a constraint for Governments to scale up the key health interventions in some countries. We evaluate the cost effectiveness of implementing IMNCI program in India from a health system and societal perspective.

**Methods:**

We parameterized a decision analytic model to assess incremental cost effectiveness of IMNCI program as against routine child health services for infant population at district level in India. Using a 15-years time horizon from 2007 to 2022, we populated the model using data on costs and effects as found from a cluster-randomized trial to assess effectiveness of IMNCI program in Haryana state. Effectiveness was estimated as reduction in infant illness episodes, deaths and disability adjusted life years (DALY). Incremental cost per DALY averted was used to estimate cost effectiveness of IMNCI. Future costs and effects were discounted at a rate of 3%. Probabilistic sensitivity analysis was undertaken to estimate the probability of IMNCI to be cost effective at varying willingness to pay thresholds.

**Results:**

Implementation of IMNCI results in a cumulative reduction of 57384 illness episodes, 2369 deaths and 76158 DALYs among infants at district level from 2007 to 2022. Overall, from a health system perspective, IMNCI program incurs an incremental cost of USD 34.5 (INR 1554) per DALY averted, USD 34.5 (INR 1554) per life year gained, USD 1110 (INR 49963) per infant death averted. There is 90% probability for ICER to be cost effective at INR 2300 willingness to pay, which is 5.5% of India’s GDP per capita. From a societal perspective, IMNCI program incurs an additional cost of USD 24.1 (INR 1082) per DALY averted, USD 773 (INR 34799) per infant death averted and USD 26.3 (INR 1183) per illness averted in during infancy.

**Conclusion:**

IMNCI program in Indian context is very cost effective and should be scaled-up as a major child survival strategy.

## Introduction

Neonatal and infant mortality accounts for 55% and 77% of total under-5 child mortality in India respectively [1–4]. Reducing high levels of infant and neonatal mortality is the key towards achieving the fourth Millennium Development Goal (MDG-4) goal. While various measures are being undertaken by Government of India for reducing child mortality, specific focus remains on improving neonatal and infant survival. Against this backdrop, Integrated Management of Childhood Illnesses (IMCI) was implemented in India after being adapted as Integrated Management of Neonatal and Childhood Illnesses (IMNCI) [5]. Specific focus is laid on early newborn care through home visits and improving home-based newborn care practices, besides upgrading skills of health workers and doctors for managing sick children at health facilities. It also aims at strengthening of health systems through better availability of drugs and personnel, and provision of referral services [6].

Review of evidence on the impact of Integrated Management of Childhood Illnesses (IMCI) shows that it is associated with reduction in childhood morbidity [7]. Trials from Tanzania and Brazil indicate lesser morbidities among the children who lived in areas where the IMCI program was implemented [8–10]. Similarly, studies from Uganda and Bangladesh show better performance of health workers trained in IMCI in diagnosing and managing sick children [9,11]. Studies on effect of IMNCI on childhood mortality have been less conclusive. While a 13% reduction in childhood mortality was found in Tanzania, no significant difference in mortality was observed in Brazil or Bangladesh. A recent cluster-randomized trial from district Faridabad in Haryana state of India reported a 15% (6% to 23%) reduction in infant mortality (adjusted hazard ratio of 0.85) as a result of IMNCI program [12]. Similarly, reduction in prevalence of pneumonia and diarrhoea was modelled using estimates from the IMNCI effectiveness trial [13]. The adjusted risk ratios reported for pneumonia and diarrhoea among infants with IMNCI were 0.73 (0.52, 1.04) and 0.71 (0.60, 0.83), as compared to a setting of routine care without IMNCI.

Despite evidence base on impact of IMCI, Child Survival Countdown conference in 2005 noted lack of financial resources as a constraint by Governments in scaling up the key health interventions in some countries [7]. India introduced the IMNCI program in 2002 on a pilot basis in 6 districts. Currently IMNCI is being implemented in 433 districts out of a total of 640 districts [14].

Although IMCI program has been evaluated from an economic viewpoint in Africa [8] and Bangladesh, however, no economic evaluation of the IMNCI program has been reported in India. Operational differences in the way IMNCI is implemented vis a vis IMCI make it difficult to generalize results from such economic evaluations from Africa to India. Secondly, results from previous economic evaluations of IMCI program have been limited to cost comparisons, while an overall incremental cost effectiveness analysis is lacking [15,16]. In the present paper we analyze the incremental cost effectiveness of implementing IMNCI at district level against a comparator of routine child health services. We report outcomes from both health system and societal perspective.

## Methods

### Ethical Approvals

The ethics review committees of the Society for Applied Studies IRB00001359) and the World Health Organization, Geneva (05015NCH) approved the study. Permissions were obtained from state and district authorities, community leaders, and women under surveillance. Women with a live birth gave informed consent before the first interview. A Study Advisory Group and a Data Safety Monitoring Board provided oversight to the study.

### Background of IMNCI Intervention Trial in India

One of the northern states, Haryana falls in the top quartile in terms of per capita gross domestic product (GDP) in India. Overall population of the state is almost 25 million [17]. The Human Development Index (HDI) value of the state is 0.545 [18]. Considering the human and economic development in the state, it is surprising that for infant mortality rate (42 per 1000) Haryana ranks 27 among 35 states and Union Territories in India.

In 2002, IMNCI was first implemented in 6 districts of India on pilot basis. Its implementation was scaled-up over the years to cover 433 districts in 2012. Considering the need for building evidence on effectiveness of IMNCI for reducing neonatal and infant mortality in India, a cluster randomized trial was planned in district Faridabad of Haryana state. The catchment area of 18 primary health centres (PHCs) was used as clusters for randomization. Prior to the trial, a baseline study was done in 2006 to assess basic characteristics and level of neonatal mortality in the 18 PHCs of Faridabad district. These 18 PHCs were stratified into 3 parts based on the baseline neonatal mortality. Three PHCs were randomly selected from each of these 3 strata for implementation of IMNCI program, while the remaining PHCs continued to implement routine child health services. More details on study design are available elsewhere [12]. Despite being contiguous, the way health institutions are organized and job responsibilities defined for health workers, there is little possibility for risk of contamination.

A cluster randomized trial to assess the impact of IMNCI was conducted from January 2007 to April 2010 in district Faridabad of Haryana state in India [12]. Impact was assessed in terms of reduction in infant mortality, infant morbidity (i.e. pneumonia and diarrhoea) and neonatal severe illness (Table 1). IMNCI program was implemented in 9 primary health centres (PHCs), with a total population of 537915. Remaining 9 PHCs, which catered to 587,213 population of Faridabad district, implemented routine child health services without IMNCI. A pregnancy surveillance was done in which 77587 pregnant women were identified and registered in the population under 18 PHCs (intervention and control arm) of district Faridabad. Outcome of the pregnancy was captured during the period from 2008–2010 through multiple contacts. A newborn cohort was identified based on the baseline pregnancy surveillance. This newborn cohort was followed up at 2 time points i.e. 6 months and 1 year from 2008 to 2010. Mothers remained the respondent in these surveys conducted at different time points. The intervention included training the frontline workers (auxiliary nurse midwife (ANM), child care workers or *anganwadi* workers (AWW) and accredited social health activists (ASHA)) on clinical algorithms for management of sick children. This enhanced their skills to assess, diagnose and treat a sick child. Besides the health workers, orientation sessions were conducted for traditional birth attendants and registered medical practitioners. AWW also performed home visits for newborn care. Newborn and child care practices at household level were improved through a behaviour change communication campaign involving women group meetings, installation of wall paintings and banners.

**Table 1:** Demographic, Epidemiological and Impact Parameters for IMNCI Cost Effectiveness Decision Model.

Parameter	Base Value	Lower Limit	Upper Limit	Source
**Demographic Parameters**				
Total Population (Rural)	1188654	950923	1426385	RGI (2011) [47]
Birth Rate (per 1000 population)	25.7	20.56	30.84	DLHS-3 [3]
Annual Growth Rate (%)	3.17	2.54	3.80	IIPS (2006) [4]
Decline in Birth Rate (%)	1.5	1.2	1.8	IIPS (2006) [4]
Infant Mortality Rate (per 1000 live births)	41.70	33.36	50.04	Bhandari N et al (2012) [12]
**Epidemiological Parameters**				
Severe neonatal illness/Danger signs (%)	20.6	16.48	24.72	Mazumder S et al (2014) [13]
Morbidity rate for pneumonia, Infant (%)	12.30	9.84	14.76	Mazumder S et al (2014) [13]
Morbidity rate for diarrhoea, Infant (%)	28.40	22.72	34.08	Mazumder S et al (2014) [13]
Average length of Illness	8.15	6.52	9.78	Sharma AK (1999) [48]
**Impact Parameters**				
Reduction in Infant Mortality with IMNCI (hazard ratio)	0.85	0.77	0.94	Bhandari N et al (2012) [12]
Reduction in Pneumonia among Infants with IMNCI (adjusted risk ratio)	0.73	0.52	1.04	Mazumder S et al (2014) [13]
Reduction in Diarrhoea among Infants with IMNCI (adjusted risk ratio)	0.71	0.60	0.83	Mazumder S et al (2014) [13]
Reduction in neonatal severe illness/Danger signs (adjusted risk ratio)	0.82	0.67	0.99	Mazumder S et al (2014) [13]

Health system was strengthened in the IMNCI area through triple interventions. Availability of essential medicines such as oral rehydration solution (ORS), zinc, co-trimoxazole, paracetamol and gentian violet paint was improved at the level of health workers, *anganwadi* workers (AWW) and ASHA through creation of village level drug depots. Sick newborn care unit (SNCU) was established at the district hospital with facilities for radiant warmers, neonatal resuscitator, vital sign monitor, pulse oximeter, infantometer, phototherapy unit and oxygen to provide level-II newborn intensive care [19,20]. Similarly, newborn stabilization units were established at three First Referral Units (FRUs).

Secondly, supervision and monitoring of program was strengthened with contractual recruitment of staff to fill vacancies. Supervisory visits focused on performance of home visits, assessment and reinforcement of clinical and counselling skills of health workers, ‘retraining’ of skills in which heath workers were deficient, review of facility support and promotion of community IMNCI through the existing staff. Lastly, task-based incentives were provided for IMNCI work. ASHAs routinely get incentives for promoting institutional births (INR 100, US$ 2.3) and immunization (INR 100, US$ 2.3). In the intervention clusters, they received additional incentives for conducting postnatal home visits (INR 75, US$ 1.7), treating sick newborns and children (INR 35, US$ 0.8), and conducting women's group meetings (INR 35, US$ 0.8).

#### Routine child health services

Under the control scenario, all services under the ambit of Reproductive and Child Health (RCH) program were delivered [21]. The major difference between intervention and control scenario was presence of training of health workers, more intense Behaviour Change Communication (BCC) for appropriate home-based newborn care practices, enhanced supervision of grassroot level CHWs and health system strengthening in IMNCI area.

### General Model Description

**Fig 1:**
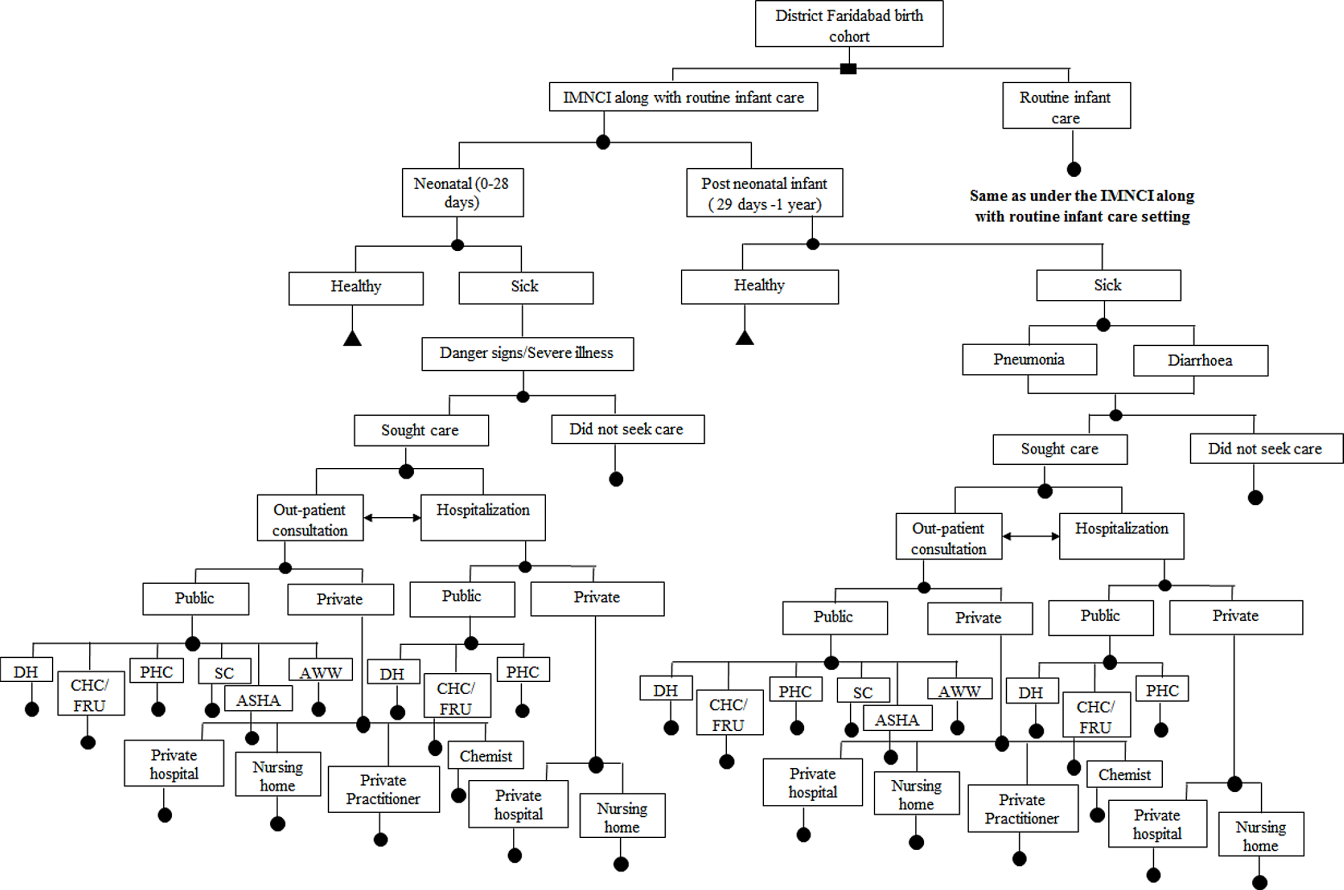
Decision Model for Cost-effectiveness of IMNCI in Haryana, India. **Note**: DH = District Hospital, CHC = Community health centre, FRU = First referral unit, PHC = Primary health centre, SC = sub-centre, ASHA = Accredited social health activist, AWW = Anganwadi worker. Cycle repeated for 15 birth cohorts (2008–2022).

**Fig 2:**
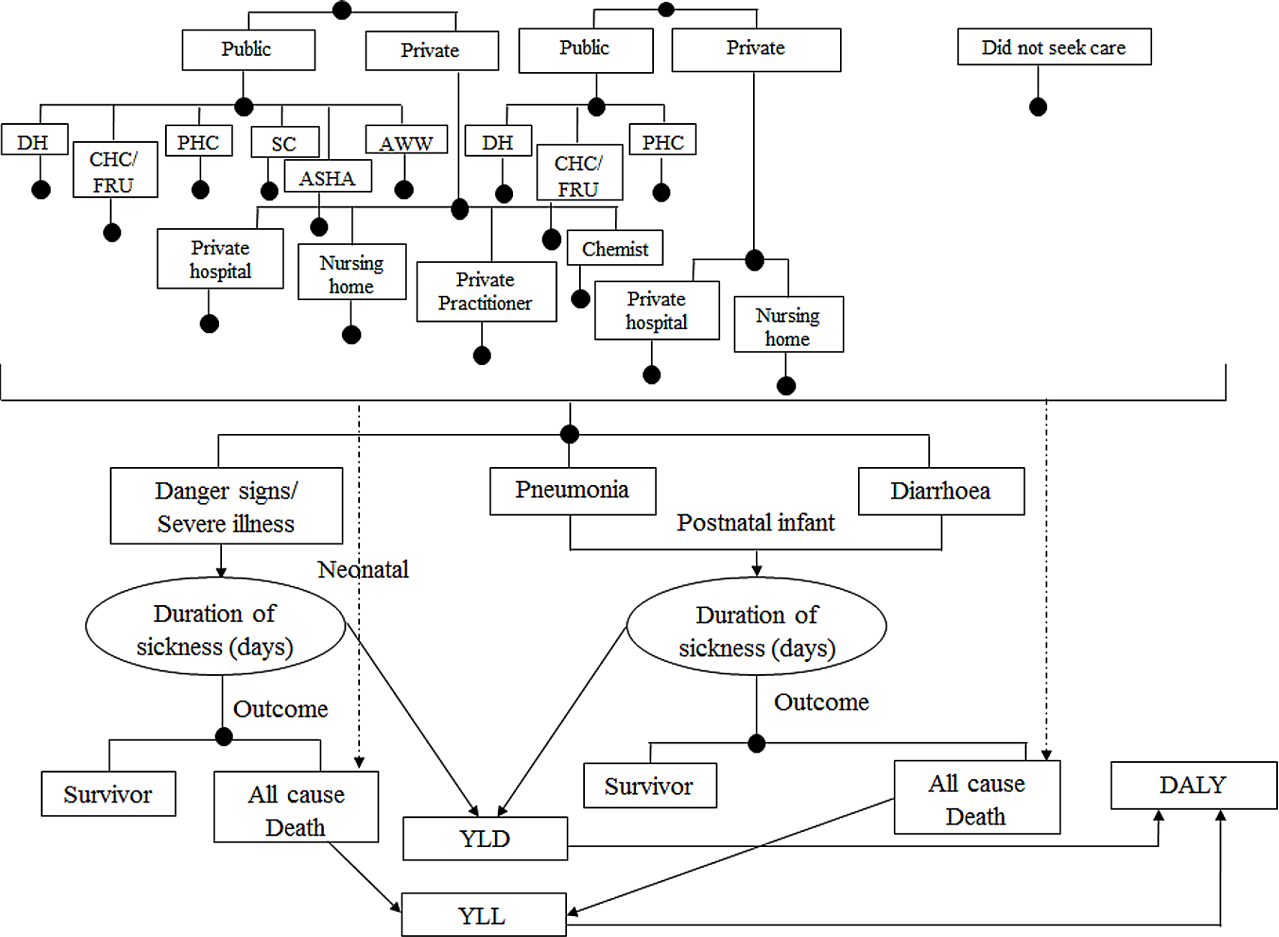
Outcome Model for Cost-effectiveness of IMNCI in Haryana, India. **Note**: In continuation to the model 1, outcome model describes the probable scenarios after a neonatal or post-neonatal infant had been treated or not (irrespective of type of health facility). YLD = Years of life lived with disability, YLL = Years of life lost due to premature mortality, DALY = Disability adjusted life years.

A decision tree was parameterized on MS-Excel spreadsheet to estimate the incremental cost effectiveness of implementing IMNCI program (Figs 1 and 2). A time horizon of 15 years starting from base year of 2007 was considered appropriate to cover all costs and effects comprehensively. Ideally, period of time horizon should be such that it covers all important costs and consequences as a result of the intervention. In the case of IMNCI, while several costs of capital nature are incurred during the early years of implementation, however, the consequences of those investments continue to occur till many years later. For example, the improvements in care seeking for childhood illnesses continue to be exercised by households even after once the costs on behaviour change communication are incurred. Similarly, benefits such as reduction in morbidity as a result of increased coverage of preventive interventions such as immunization and better home care practices continue to occur for several years till the children are susceptible to the vaccine preventable diseases, or other communicable diseases. Most of these illnesses have a predominant risk within the first 5 years of life, with gradually declining risk till about 15 years. As a result previous cost-effectiveness studies evaluating child health interventions in India chose to use a time horizon varying from 5 years to lifetime study horizon [22]. The reference case analysis recommended by WHO suggests using a lifetime study horizon [23]. In view of these arguments we consider 15 year time horizon appropriate. We analyzed costs and effects from both health system and societal perspective. Effect was measured in terms of illness episodes averted, child deaths prevented, life years gained and disability-adjusted life years (DALY) averted. Both costs and effects were discounted at 3% to account for time preference of cost and utility. We estimated the standardized unit cost from health system and societal perspective. We report our findings as incremental cost of implementing IMNCI for infants per DALY averted, per illness episode prevented and per infant death averted as compared to routine care services [24].

### Cost Data Collection and Analysis

Health system costs of providing services through district hospital, community health centre (CHC), ANM, AWW and ASHA was ascertained using a bottom-up ingredients approach [25]. This approach begins by defining the type and quantity of input or ingredients used to produce the service output. The input may include capital and recurrent resources. The price of each type of input (or ingredient) used is ascertained. Next, the number of each input unit used is multiplied by the prices to obtain the overall cost of the input used. Finally, costs of all input are summed and divided by the number of products produced to obtain the unit cost for each product or “output” of that service. We randomly selected 8, 9 and 8 auxiliary nurse midwives (ANMs), *anganwadi* workers (AWWs) and accredited social health activists (ASHAs) respectively from areas implementing IMNCI program in district Faridabad in North India; and 10, 9 and 10 ANMs, AWWs and ASHAs respectively, from areas in the same district not implementing IMNCI. Health system costs of delivering child health care services to under-5 years children by the primary care workers, was assessed in both the alternative scenarios of with and without IMNCI. One FRU and the district hospital were also included for costing. Detailed methods on data sources, methods of apportionment for child health, and analyses undertaken are available elsewhere [26] and in supplementary appendix (Appendix A in S1 Material). Cost of delivering child health services at PHC was obtained from estimates reported in the WHO-CHOICE study [27].

Treatment seeking behaviour and out-of-pocket (OOP) costs given in Tables 2 and 3 were estimated by analyzing data from the Indian IMNCI trial [12]. Out-of pocket costs include money spent on medicines, diagnostics, consultation charges paid to doctor, bed-charges paid for admission, procedures, transportation to health facility, and loss of productivity on account of the parents’ absence from work. Productivity loss was derived based on the monthly salary and the number of days spent away from work for child care. Mothers of 6204, 3072 and 2042 children at the age of 29 days, 6 months and 12 months respectively were interviewed from the intervention area. Similarly a total of 6163, 3047 and 2014 mothers with children at the age of 29 days, 6 months and 12 months respectively were interviewed from the control area. These mothers were interviewed for any episode of diarrhoea or pneumonia during last 15 days (at 6 and 12 months) and any illness and its symptoms (at 29 days), the type of provider where treatment was sought and OOP costs at each provider level (Table 2). Number and type of multiple providers sought for a specific illness was also analyzed.

**Table 2:** Treatment Seeking Behaviour Parameters for IMNCI Cost Effectiveness Decision Model.

Utilization Parameters	Intervention			Control		
OPD Treatment	Base Value	Lower Limit	Upper Limit	Base Value	Lower Limit	Upper Limit
District Hospital	0.011	0.008	0.015	0.026	0.021	0.031
Primary Health Centre	0.005	0.003	0.007	0.012	0.008	0.015
AWW	0.132	0.121	0.143	0.002	0.001	0.004
ASHA	0.046	0.039	0.053	0	0	0
Chemist	0.045	0.039	0.052	0.074	0.066	0.081
Private Practitioner	0.559	0.543	0.574	0.712	0.699	0.726
Nursing Home	0.089	0.08	0.098	0.065	0.058	0.073
Private Hospital	0.022	0.018	0.027	0.052	0.045	0.058
ANM	0.001	0.004	0.009	0.001	0.004	0.009
**IPD Treatment (Hospitalizations)**						
District Hospital	0.07	0.05	0.08	0.06	0.05	0.07
Private Hospital	0.93	0.75	1.12	0.94	0.75	1.13

**Source:** *Estimates for treatment seeking behaviour given in the table are based on the author analysis of primary data collected under India IMNCI Impact RCT study [6].*

**Table 3:** Health system and out-of-pocket (OOP) Cost Parameters for IMNCI Cost Effectiveness Decision Model.

Cost of Health Care*	Intervention			Control		
*Per Infant costs*	Base Value	Lower Limit	Upper Limit	Base Value	Lower Limit	Upper Limit
General health system administration	22	17	26	22	17	26
Program cost	57	46	68	-	-	-
***Per Infant OPD cost***						
District Hospital	578	462	694	578	462	694
First Referral Unit	274	219	329	274	219	329
Community Health Centre (CHC)	274	219	329	274	219	329
Primary Health Centre (PHC)	178	143	214	178	143	214
AWW	602	482	723	573	459	688
ASHA	110	88	132	78	62	93
Subcentre	435	348	522	339	271	407
***Per Infant IPD cost***						
District Hospital	3854	3083	4625	3854	3083	4625
First Referral Unit (Intervention)	1828	1463	2194	1828	1463	2194
Community Health Centre (CHC)	1828	1463	2194	1828	1463	2194
Primary Health Centre (PHC)	1189	951.2	1426.8	1189	951	1427
***Per Infant OOP cost for OPD***						
District Hospital	119	61	178	89	63	115
PHC	9	0.0	18.07	34.7	4.2	65.2
AWW	0.08	0.01	0.15	0.6	0.0	1.8
ASHA	0.42	0.0	1.16	0.0	0.0	0.0
Chemist	30	26	33	34	31	38
Private Practitioner	104	97	110	111	105	117
Nursing Home	310	257	363	249	206	293
Private Hospital	351	275	427	323	279	368
Subcentre	3.7	0.4	6.9	16.3	8.1	24.5
***Per Infant OOP cost for IPD***						
District Hospital	1900	1265	2535	1394	811	1977
Private Hospital	5365	4887	5843	5141	4328	5954

**Source 1:** *Out-of-pocket (OOP) cost estimates (OPD and IPD) given in the table are based on the author analysis of primary data collected under India IMNCI Impact RCT study [6].*

**Source 2:** *Health system cost estimates per infant (OPD and IPD) at different levels of facilities is based on author analysis of primary cost data collected from district Faridabad under this study*.

* The unit for costs given in the table is INR (i.e. Indian National Rupee). All costs were converted to 2009 prices.

We segregated health system costs in the intervention area as those for healthcare service delivery, program implementation and general health administration. Control area health system costs were divided into healthcare service delivery and general health administration. Service delivery costs comprised of costs incurred at the level of health facilities to deliver the preventive and curative services. General health administration for child health includes costs of monitoring and evaluation, supervision, information education and communication (IEC), trainings entirely or partially for child health other than IMNCI. Resources in various forms are consumed at all levels including building, space, equipment, furniture, human resources, medicines, other consumables, and overheads such as water, electricity, laundry etc.We also estimated the cost for delivering IMNCI program in terms of costs of IMNCI trainings, capital infrastructure, human resources, social mobilization, IEC etc. incurred specifically for program implementation (S1 Material, S1 Dataset). We assumed the life of a training to be 7 years and therefore dealt with the training cost as capital in nature at time of analysis. The life of training was considered to be 7 years, based on a need for retraining health workers after an interval of 7 years. This was based on consultation with the program implementers.

The health system and OOP costs at each level of service provision, along with the morbidity rate and probability of seeking treatment at a given provider were used to estimate the standardized total cost of curative care for infant population at district level using demographic rates and health system characteristics of district Faridabad as standard district (Tables 1–3). Preventive services, costs of monitoring and support from district level including cost of health system strengthening and training costs were also added to arrive at total cost of infant health care services (Table 3). The standardized cost of infant care services (overall and unit costs) were estimated at district level for both the comparator scenarios i.e. routine services plus IMNCI and routine care. Detailed methods for costing are available in supplementary material (Appendix A in S1 Material).

### Data Sources and Analysis for Estimation of Effects

#### Intervention Setting: IMNCI

For intervention setting, findings from the Indian IMNCI effectiveness trial on infant and neonatal mortality and morbidity were used to estimate the impact of IMNCI [6]. We interpolated the impact of IMNCI i.e. reduction in mortality and morbidity in intervention area from 2007 to 2010 using logarithmic distribution. For future years, we assumed no further reduction in mortality and morbidity beyond the trial period i.e. from 2010–2022 but we assumed sustained levels of mortality and morbidity achieved as an effect of IMNCI. Valuation of consequences of implementation of IMNCI was done for its effect on infant morbidity and mortality. Impact of IMNCI on morbidity in the trial was seen only on the most common illnesses i.e. Diarrhoea and Pneumonia for infants; and severe illness/danger signs in neonatal age group. Thus, we also assumed no impact of IMNCI in reducing morbidities other than diarrhoea, pneumonia in infants and severe illness/danger signs in neonates for our effectiveness model. This was considered appropriate as there is no local evidence on impact of IMNCI on reduction of other illnesses. For impact of IMNCI on infant mortality, the trial recorded all deaths which occurred during the infant age group. As a result, this included effect of IMNCI on neonatal and infant mortality irrespective of cause. Finally, our assumptions are on a more modest note in terms of current and future effects. We assumed reduction in infant mortality of 15% (6% to 23%) with IMNCI using the estimates of India IMNCI effectiveness trial [12]. For morbidity, we imputed reduction in diarrhoea ((adjusted risk ratio 0.71 (0.60,0.83) and pneumonia (adjusted risk ratio 0.73 (0.52, 1.04) as reported in the trial [13]. (Table C in S1 Material). For reduction in morbidity among infancy, the IMNCI trial measured outcomes by following up infants at 29 days, 6 months and 1 year age and enquired about any illness in the last 2 weeks. Consequently, impact of IMNCI on reduction of illnesses at an age of 29 days was assumed as the corresponding reduction during neonatal period in our model. For post-neonatal infancy period, observations on reduction on pneumonia and diarrhoea were available at 6 months and 12 months age. We populated our model using the effectiveness estimates from trial to project reduction in diarrhoea and pneumonia among post-neonatal infants, using reductions reported at 6 months follow-up during the IMNCI trial. Two factors favoured the choice of use of 6-month observation from trial. Firstly, the 6-month observation falls in the centre of the infancy period; and secondly, the reductions in diarrhoea (29%) and pneumonia (27%) episodes at 6 months were more modest than corresponding reductions at 1 year follow-up (37% for diarrhoea and 40% for pneumonia).

#### Routine infant health care services

Baseline estimates from IMNCI effectiveness trial were used for infant mortality and morbidity in the control setting of our model [12,13]. For control area with routine health care services, estimates of infant mortality rate from three different rounds of National Family Health Survey (NFHS) i.e. first in 1992–93, second in 1998–99 and third in 2005–06 were interpolated using logarithmic estimations from the year 1992 to 2006. Same estimated rate of reduction for IMR was used and extrapolated from 2007 to 2022. In order to compute the number of illness episodes among children, we used the proportion of children for whom commencement of an illness episode was reported during last 15 days period during the NSS survey [3,4].

#### Valuing effects: primary and secondary endpoints

Primary endpoint for estimation of effects in our study is reduction in disability adjusted life years (DALY). DALY is a sum of years of life lost (YLL) as a result of premature mortality and years of life lived in disability (YLD) [28]. For calculating YLL in case of an infant death, we estimated that the mean age of infant death is 26 days. This estimation was based on the assumption that 60% of infant deaths occur in neonatal period, 60% of neonatal deaths are early neonatal deaths (within first 7 days of birth) [1,29,30]. We also assumed that mean age of early neonatal, late neonatal and post-neonatal death is 3 days, 20 days and 6 months respectively; i.e. the mid-point of class interval. We calculated YLDs separately for each of the three most common illnesses in infancy (Tables A-B in S1 Material). Subsequently, YLDs were estimated for complications as a result of the childhood morbidities (Table C in S1 Material). We used disability weights reported in the Global Burden of Disease (GBD) estimates [31].

### Sensitivity Analysis

We undertook univariate sensitivity analysis to assess the influence of uncertainty in each parameter on the overall value of ICER [24]. We tested our assumption of logarithmic distribution for smoothing in trends of mortality and morbidity, by comparing ICERs computed using a logarithmic distribution against that without any logarithmic smoothing in trends of mortality and morbidity.

We tested our assumption of future effects used in the base estimate with a best and worst case scenario using the 95% confidence limits (CI) of estimates reported from Indian trial [12]. In the best case scenario, lower bounds of CI for costs and higher bounds of CI for relative decline in mortality rate, morbidity rate were assumed, whereas, in contrast, higher bounds of CI for costs and lower bounds of CI for relative decline in mortality rate, morbidity rate were assumed, for worst case scenario. Future effects beyond 2009 were similar as in the base case.

Finally, we undertook a probabilistic sensitivity analysis (PSA) to estimate the effect of joint uncertainty in all parameters. Probability of IMNCI program to remain cost effective at a willingness to pay threshold equal to per capita gross domestic product (GDP) was estimated, using a health system perspective. For undertaking PSA analysis, we used log-normal distribution for cost parameters and parameters for reduction in morbidity and mortality; and beta distribution for parameters related to care seeking behaviour. For rest of the parameters we used uniform distribution to simulate random values, after assuming an upper and lower bound which were 20% on either side of base estimate. For parameters with no ranges available from any source, we assumed an upper and lower limit by varying the base value by 20% on either side of base value. Monte Carlo method was used for simulating the results over 999 times. Average costs, effects and cost-effectiveness ratios were computed along with 2.5^th^ and 97.5^th^ percentile to estimate 95% confidence interval.

## Results

### Base Results

We found that implementation of IMNCI at district level in India yielded effects in terms of averting 57861 illness episodes, 1967 deaths and 63249 million DALYs among infant population during a 15-year period from 2007 to 2022 (Table 4). The averted infant deaths lead to an increase in the total life years by 0.13 million years after age weighting. Cumulatively, IMNCI program as compared to routine services alone, results in about 0.06 (0.04–0.15) million reduction (13% relative reduction) in DALYs among infants (Table 4).

**Table 4:** Cost, Effects and Cost-Ef fectiveness of IMNCI Program in India.

Characteristics*	Base case	LL	UL	Best case	Worst case
**Cumulative Health System Cost (In Millions INR) **					
*With IMNCI*					
Service delivery	555	387	745	353	794
General health system administration	9	6	12	6	13
Program cost	24	17	32	15	34
Overall	589	410	790	375	842
*Without IMNCI*					
Service delivery	481	335	653	305	572
General health system administration	9	6	12	6	11
Overall	491	342	666	312	584
*Incremental health system cost with IMNCI*	98	69	124	63	258
**Cumulative Societal Cost (In Millions INR) **					
With IMNCI	657	460	896	414	931
Without IMNCI	588	408	807	364	700
*Incremental societal cost with IMNCI*	68	52	89	50	230
**Standardized Health System Cost Per infant (INR)**					
With IMNCI	1231	1097	2112	984	1477
Without IMNCI	1022	913	1781	816	1022
*Incremental health system cost with IMNCI*	209	184	331	168	455
**Standardized Societal Cost Per infant** **(INR)**					
With IMNCI	1368	1230	2396	1083	1627
Without IMNCI	1226	1090	2158	954	1226
*Incremental societal cost with IMNCI*	142	139	238	129	402
**Incremental Effects with IMNCI**					
Infant Illness Episodes prevented	57861	30702	58356	65937	57288
Infant Deaths averted	1967	1213	4343	4219	2449
Life Years added	130825	40599	152165	233354	163093
DALY averted	63249	40583	152169	135719	78742
**Incremental cost effectiveness ratio, health system perspective **					
Cost per illness averted	1699	807	4210	956	4504
Cost per infant death averted	49963	13923	96274	14943	105340
Cost per DALY averted	1554	428	3018	465	3277
**Incremental cost effectiveness ratio, Societal**					
Cost per illness averted	1183	0	3804	751	4022
Cost per infant death averted	34799	0	80996	11743	94075
Cost per DALY averted	1082	0	2460	365	2926

* INR—Indian National Rupee; LL–lower limit; UL–upper limit.

Note: All the estimates in the table represent the year 2009.

**Fig 3:**
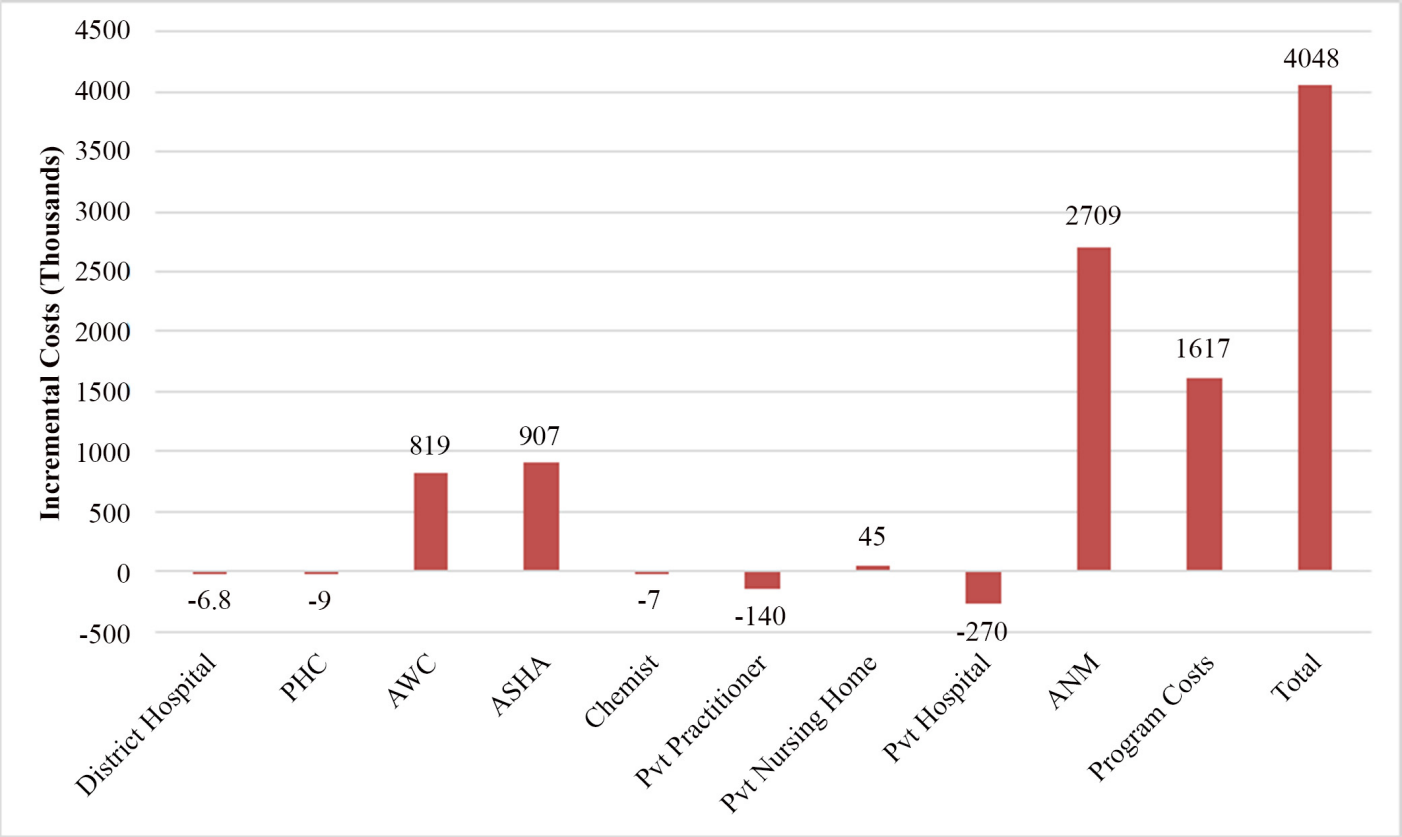
Change in costs at different levels on account of IMNCI program.

**Fig 4:**
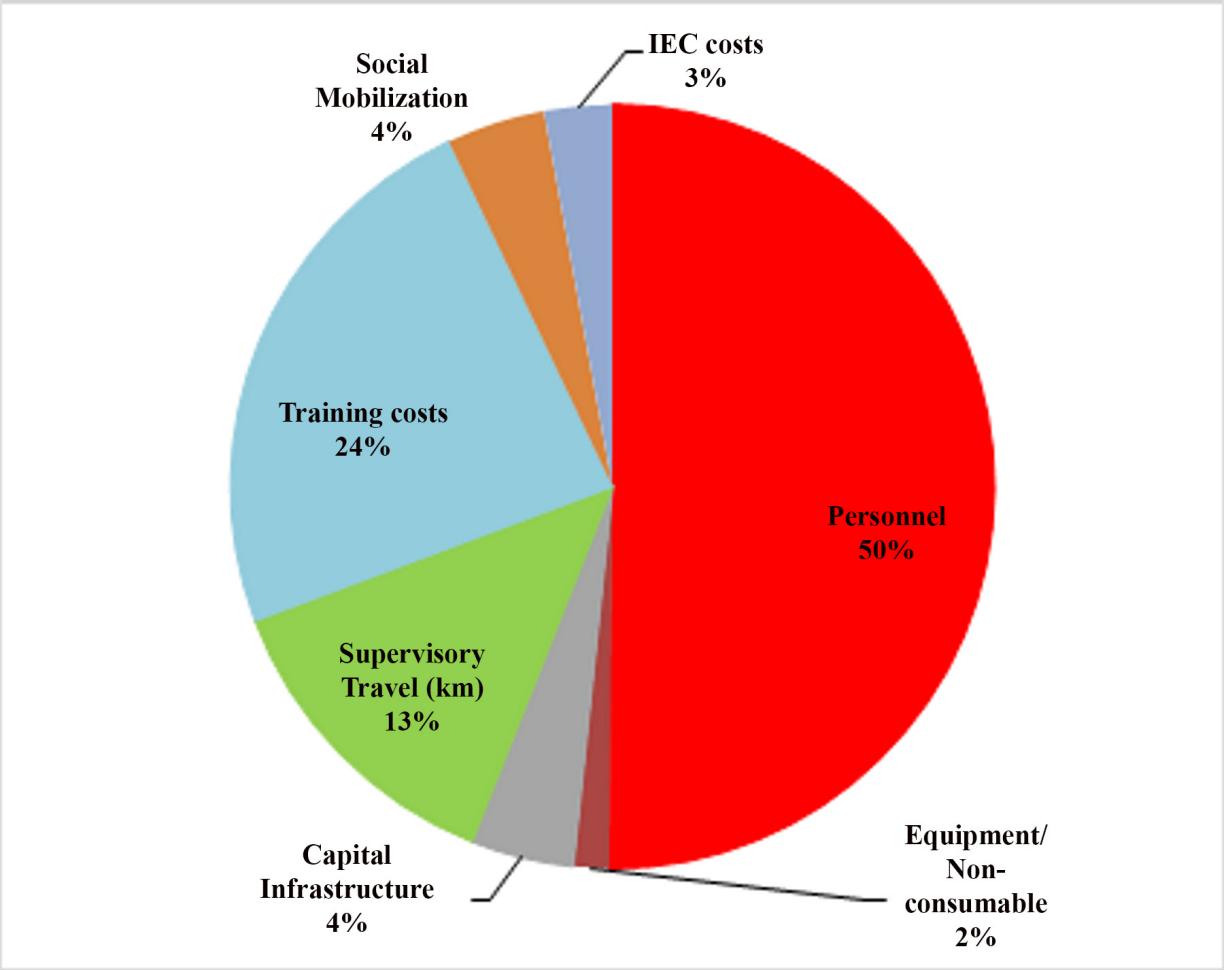
Distribution of incremental program implementation and monitoring costs as a result of IMNCI.

In terms of cost, we found that the health system’s standardized cost per infant for implementation of routine child health services was INR 1022 (USD 22.7). With IMNCI, delivery of child health services costs INR 1231 (USD 27.4) per infant to the health system. Overall, it implies that IMNCI incurs an additional cost of USD 2.2 million (INR 98 million) to the health system from 2007 to 2022. This incremental health system cost was on account of increased service delivery for delivering additional services due to change in care seeking and program costs for implementing IMNCI program. Three-fourth of the total incremental costs of IMNCI implementation at district level is explained by increased utilization of healthcare services at primary care level i.e. ANM, AWW and ASHA (Fig 3). IMNCI program implementation costs account for the one-fourth of incremental costs. This is incurred on account of trainings of healthcare personnel, augmentation of drug and supplies and strengthening of monitoring and supervision system. Half of the incremental costs of IMNCI implementation is on accounts of personnel costs (Fig 4).

From a societal viewpoint, the incremental cost of IMNCI was USD 1.5 million (INR 68 million). The societal incremental costs were less than the health system’s costs on account of savings in OOP spending as a result of reduction in illness episodes.

Overall, from a health system perspective, IMNCI program incurs an incremental cost of USD 34.5 (INR 1554) per DALY averted, USD 34.5 (INR 1554) per life year gained, USD 1110 (INR 49963) per infant death averted and USD 38 (INR 1699) per illness averted during infancy. Similarly, using a societal perspective, IMNCI program incurs an additional cost of USD 24.1 (INR 1082) per DALY averted, USD 773 (INR 34799) per infant death averted and USD 26.3 (INR 1183) per illness averted in during infancy (Table 4).

### Sensitivity Analysis

We found that effectiveness of IMNCI in terms of reduction in infant morbidity and mortality; health system costs at *anganwadi* level and at subcentre level, baseline incidence of infant morbidity and total population had highest influence on the incremental cost per DALY averted.

**Fig 5:**
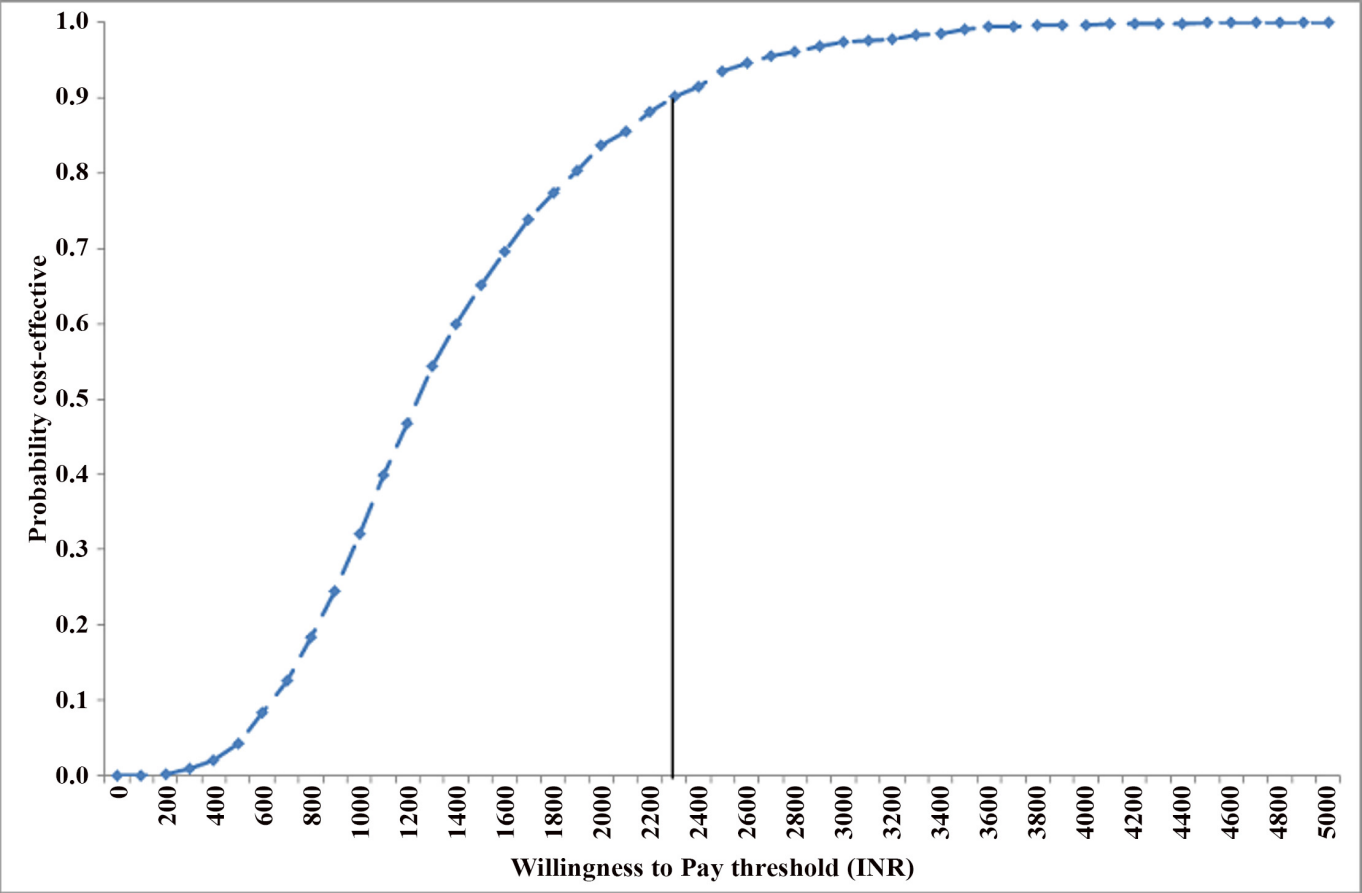
Probability of IMNCI Program to be cost effective at varying willigness to pay thresholds per DALY averted.

As a part of sensitivity analysis, we did a best and worst case scenario analysis varying the parameter values to both extremes. In the best case scenario, where high infant mortality and morbidity rates and; low implementation cost was assumed, we found an incremental cost of USD 10.3 (INR 465) and USD 8.1 (INR 365) per DALY averted from a health system and societal perspective respectively. In the worst case scenario, where we assumed low infant mortality and morbidity rates and; high implementation costs, we found an incremental cost of USD 72.8 (INR 3277) and USD 65 (INR 2926) per DALY averted from a health system and societal perspective respectively (Table 4). In both these contrasting scenarios, IMNCI remains very cost effective in India. Finally, we found that the IMNCI program has a more than 90% probability of being cost effective from a health system perspective at willingness to pay threshold equaling INR 2300 (USD 51) per DALY averted, which is 5.5% of India’s GDP per capita. (Fig 5, Fig A in S1 Material).

## Discussion

We undertook the present economic evaluation to compare the costs and consequences of implementing IMNCI for infant population, compared to routine child health services. In our analysis, we report findings from both a health system and societal perspective. The approach suggested by the commission for Macroeconomics on Health (2001) is that interventions with an incremental cost per DALY averted less than the per capita GDP in low middle income countries (LMICs) are “very cost effective”, and those costing less than triple the per capita GDP are “cost-effective”. India had a GDP per capita of INR 67635 (USD 1503) in 2012 [32]. Our analysis shows that IMNCI implementation costs the Government of India an additional USD 34.5 (INR 1554) per DALY averted. There is 90% probability for IMNCI program to be a cost effective strategy from a health system perspective at a willingness to pay threshold of USD 51 (INR 2300). An intervention is considered as “very cost-effective” if the incremental cost-effectiveness ratio is less than the per capita gross-domestic product; “cost-effective” if it is between 1–3 times per capita national GDP; and “not cost-effective” if the value of ICER is more than 3 times the GDP per capita. In the case of IMNCI, the value of ICER was less than the GDP per capita in all simulations as part of the probabilistic sensitivity analysis. We found more than 90% probability that the value of ICER is less than INR 2300 willingness to pay, which is 5.5% of India’s GDP per capita in 2009. Our results show that IMNCI program is similar in terms of its economic value to some of the most cost effective child health interventions reported for developing South East Asian countries [33].

Our study findings point to the changes in health system and out-of-pocket costs brought about as a result of the IMNCI program. Program implementation and monitoring explains only 25% of the overall increase in health system costs. Remaining costs were incurred on account of the increased demand for health services in public sector. This increased demand was a manifestation of 2 factors–firstly, better care seeking for illnesses which were earlier not treated, and secondly, on account of a shift in care seeking which resulted in a switch from private to public sector and from higher levels of care to primary care providers. The latter finding is also significant as it resulted in a reduction of the out-of-pocket expenditures for treatment. This explains the lower ICER from a societal perspective than the health system perspective, as the former accounts for reduction in out-of-pocket expenditure.

To our knowledge, this study is the first attempt from an Asian country to systematically ascertain the cost effectiveness of IMNCI program. A number of estimates for cost effectiveness of individual child health interventions are available such as haemophilus influenza type ‘b’ vaccine [34], insecticide treated bednets for malaria [35,36], HIV preventive interventions for maternal to child transmission etc [37]. Barring a study from Bangladesh, no previous economic evaluation from a South Asian country has documented the costs and effects of delivery of a comprehensive child health program covering the entire gamut of preventive and curative services [38]. However, even the Bangladesh study restricts its focus to the costs and outcomes during the neonatal age group. Another previous economic analysis for IMCI from Bangladesh has been undertaken, however, it is restricted to assessing additional resource requirements for IMCI, mainly in terms of community health workers [15]. Other studies from Tanzania, Brazil and Uganda have found that IMCI program is either cost saving or cost neutral [10,39,40]. Our study also merits to being the first one which has found the effects in the standard terms of disability adjusted life years, which facilitates in making comparisons across programs and sectors [41]. There are several studies which have evaluated the cost effectiveness of child health interventions. In terms of findings from developing country context, IMNCI program is as cost effective as some of the well-known child health interventions such as vitamin A and zinc fortification, measles immunization, case management of pneumonia and oral rehydration therapy [33]. Partial reason for this finding could be that IMNCI as a package promotes several of these individual interventions. Within the literature from India, IMNCI program is as cost effective as measles and hepatitis B vaccination, and more cost effective than other vaccines against cholera, typhoid, haemophilus influenza type ‘b’ and rotavirus [22]. We have taken care to test most of our modeling assumptions in the sensitivity analysis. All our parameter values have been drawn from studies conducted either in local area or India, and for a minority few we have drawn on literature from South East Asian region [3,4,6,27,31,42].

Our study also has a number of methodological strengths. Firstly, our decision model is completely plausible in terms of program implementation design, care-seeking and health care delivery system. A decision tree was considered appropriate than any other modelling method, such as Markov model, considering the acute nature of most childhood illnesses. Secondly, almost all the values for parameters were sourced from local Indian context. Most of these parameter values have been drawn from a randomized controlled trial which enhances the internal validity of our estimations. Thirdly, we test various uncertainties using a comprehensive approach to sensitivity analyses. Methodological assumption of logarithmic distribution was found not to have much effect on our results. Uncertainties in model structure were analyzed using a best and a worst case scenario which again strengthens the conclusion of cost effectiveness of IMNCI program. Finally, the PSA analysis tests the joint parameter uncertainties. All these enhance the generalizability of our findings for the cost effectiveness of IMNCI program. In each of these sensitivity analyses, the value of incremental cost effectiveness ratio remains less than the GDP per capita, providing credence to the conclusion of our analysis about cost effectiveness of IMNCI in Indian context.

Our estimate of cost of delivering health care services through community health workers is very similar to that reported by another recent study which estimated the cost of delivering health care services through CHWs in three north Indian states [43]. Similarly, our unit estimates for treating infants at district hospitals is again very close to what has been found in an analysis including hospitals from five districts in two north Indian states [20,44].

Among the published cost-utility studies for newborn and child health reported in a systematic review, the evaluation from Bangladesh is closest to our study in terms of the intervention design [41]. The Bangladesh study evaluated cost effectiveness of two neonatal care services packages i.e. home and community based neonatal care with the routine services in Bangladesh. The analysis was done using a societal perspective. Effectiveness was assessed using a cluster-randomized controlled trial design. The authors included program costs, health system costs and household out-of-pocket costs and reported findings in terms of incremental cost per neonatal death averted and DALY averted. Our study is similar to the Bangladesh study in terms of the nature of intervention, methodological features, reporting indicators and the analytical approach. [38]. This study found an incremental cost of USD 103 per DALY averted for the intervention as compared to routine district health system. Our estimate of ICER is lower than the Bangladesh study, which could result from a number of factors. First, the effects in the latter study were measured only among neonates, as compared to infants in our study. Secondly, there were significantly higher out-of-pocket costs in our setting which were averted as a result of morbidity reduction. This resulted in lower value of incremental cost effectiveness ratio.

### Limitations

Our study had certain limitations in terms of its design. Firstly, our model analyzes the costs and effects of IMNCI among infant population. In practice, the IMNCI program targets children less than 5 years, hence it was more pragmatic to value costs and effects in the under-5 age population. However, the trial undertaken to assess the impact of IMNCI observed effects among neonatal and infant population only. Since there were no estimates from India on the effectiveness of IMNCI on mortality and morbidity among 1–5 year age population, so we chose to restrict our model to infant population. We believe that the overall conclusion in terms of cost effectiveness of IMNCI would not change much if the costs and effects were measured in under-5 year age group. However, the value of ICER could be more in the latter scenario, as the extent of mortality and morbidity reductions are likely to be less among 1–5 year age children than the neonatal and infant age group.

Secondly, the data on health system costs was estimated as unit cost per under-5 child. Although it was ideal to estimate unit cost per infant in health system costing, however, limitations in the way data is recorded at subcentre, *anganwadi*, ASHA, CHC and district hospital level did not permit such apportioning. The services utilized in reports for each of these levels were available as either under-5 child or above-5 child, or 15–45 years old etc. Among the under-5 children, further stratification among infants and 1–5 years was not available. However, we believe that the health system costs for infants are likely to be slightly higher than the 1–5 year population, considering higher morbidity levels and higher severity of illness among infants which requires more resource intensive treatment. In view of this and the fact the IMNCI results in lowering of morbidity among infants, it can be safely assumed that IMNCI program is at least as cost effective in Indian context as found in our results.

### Conclusion

To conclude, our results show that IMNCI program is a cost effective strategy as compared to routine health care program for infant population. From a health system perspective, IMNCI is cost effective strategy for child survival in India, with a 90% probability to be cost effective at a willingness to pay threshold of INR 2300 (USD 51). Recommending a program or strategy for scale-up merely on grounds of efficiency may not be prudent. Issues of sustainability and feasibility are of paramount concern to the policy makers. At an incremental cost of INR 209 per infant per year, implementation of IMNCI imposes an incremental cost of INR 23 per capita per year. With an overall health system spending of INR 960 per capita per year [45], this implies nearly 2.4% increase in budget which appears reasonable considering the Government of India’s strong commitment to raise resource allocation to health for achieving universal health care. Sparing health care workers for 8-day long IMNCI training is again cited as a health system challenge in the context of health workforce shortage. However, an interrupted 5-day training has been shown to be equally effective and cost-effective in Indian setting [46]. Moreover, significant augmentation of community health workers under National Health Mission–India’s flagship program, will make it possible to implement the program.

## Supporting Information

S1 Dataset (XLSX)

S1 Material (DOC)
