# Supplementary material for: Cost Effectiveness of Implementing Integrated Management of Neonatal and Childhood Illnesses Program in District Faridabad, India
Source: PLoS One. 2016 Jan 4;11(1):e0145043. doi: 10.1371/journal.pone.0145043 (PMC4699694; doi:10.1371/journal.pone.0145043)

**Supplementary Material**

**Cost Effectiveness of implementing Integrated Management of Neonatal and Childhood Illnesses Program in district Faridabad, India**

Shankar Prinja, Pankaj Bahuguna, Pavitra Mohan, Sarmila Mazumder, Sunita Taneja, Nita Bhandari, Henri van den Hombergh, Rajesh Kumar

**Methodology for Costing**

We undertook an economic costing of providing health care services to infant population. As opposed to a financial costing, this method allows us to quantify and value any resource, irrespective of who pays for it. We measured the overall cost of infant health care, both curative and preventive, in two competing scenarios i.e. delivering child health services in a routine manner without IMNCI and the alternate scenario of child health care services with implementation of IMNCI. The setting of a cluster randomized trial for assessing effectiveness of IMNCI was used for the purpose of estimations. All costs at each facility level along with utilization of services were applied to a standardized district, which in our case was district Faridabad. Incremental cost of implementing IMNCI at a district level was calculated as a difference of the total cost of delivering infant health care services in the scenario with routine delivery of services from that with implementation of IMNCI.

The costs were projected for a 15 years period from 2007 to 2022. All costs were converted to 2007 Indian National Rupees (INR) and US dollars (USD) using prevailing GDP deflators and a discount rate of 3%.

**Study Sample**

We undertook costing for delivery of under-five year (U-5) child health care services at one districts hospital and one FRU/CHC. Additionally we randomly selected 9 ANM’s, 10 AWW’s and 10 ASHA’s in IMNCI area. Similarly, we included 9 ANM’s, 8 AWW’s and 8 ASHA’s from control area where routine child health care services were available. District level data was collected for trainings, monitoring, supervision and other program support activities. Unit cost estimated for U-5 health care services was applied to infant population in standardized district setting. Hence, we estimated the cost of providing health care services to infant population in two scenarios i.e. with routine services and with implementation of IMNCI.

**Micro-Costing Methodology**

***Time allocation for Human resource***

Full time equivalent and time devoted to under-5 year old children treatment/ related activities was elicited for all staff members at each health facility where the costing was undertaken. For general services staff who are involved in administration of the entire health facility, we used the ratio of under-5 children attendance and the total patient attendance, to estimate their time spent on child health care. Total salary and other allowances for each staff member for under-5 child health care were thus apportioned according to their proportional time allocation to the care of former.

For primary care workers who were interviewed after implementation of IMNCI, we used two ways to estimate the time devoted on management of a single child. Firstly, we interviewed the health, anganwadi and ASHA workers regarding time taken for managing an under-5 child and compared the time devoted by those who were trained on IMNCI versus those without IMNCI training. We also interviewed the IMNCI trained primary care workers on the time which they took to examine and manage an under-5 year old child before and after being trained on IMNCI. Both the methods yielded results which were similar in terms of the incremental time taken by IMNCI trained health workers in managing per child. We also elicited the daily number of children and adults examined by the primary care workers during one week period, in three different months of January, June and September, to capture any influence of seasonality on the attendance of children at health centres/ home visits of primary care workers. Time spent in management of under-5 year old children was estimated by multiplying average daily child contacts with the average time spent on managing each child in both intervention and control area. Remaining time with the primary care workers, which is spent in maintaining records, attending meetings, travelling for home visits etc. was apportioned in accordance with the proportion of under-five population among the total beneficiaries registered with primary care workers (comprising under-five children, adolescent girls and women in reproductive age group). More details about the methods of data collection are available elsewhere (Prinja S et al, Cost of Delivering Child Health Care through Community

Level Health Workers: How Much Extra Does IMNCI Program Cost? (2013), Journal of Tropical Pediatrics). Equation 1 mentioned below describes the estimation of time cost of primary care workers for under-5 child health.

^U5^C_ij (salary)_ = {S_ij(annual)_ * 1/n *{(^U5^T_ij_ * ^U5^C_ij_) + [(n-(^U5^T_ij_ * ^U5^C_ij_)) * ^U5^P_ij_]}} ......................1

Where ^U5^C_ij (salary)_ = Annual Salary for under-5 Child health

S_ij(annual)_ = Annual salary

^U5^T_ij_ = Average time taken to treat per child (under-5)

^U5^C_ij_ = Average under-5 child contacts per day

^U5^P_ij_ = Total under-5 population/Total population = Proportion of non care provision time devoted to child health (home visits, attending meetings, writing reports etc.)

n = Number of working hours in a day

i = 1 (ANM), 2(ASHA), 3(AWW)

j = 1 (Intervention), 2 (Control)

***Capital Costs***

Capital investment is considered to be any resource which yields effects for a period of more than one year. We annualized the cost of equipments and building based on estimates of the lifespan of the capital infrastructure, using the following formula (equation 2):

^U5^C_ω (capital)_ = Σ_ω_ (A_ω_ * φ_ω_ * ^U5^P_ω_) + Σ_ωk_ {([(I_k_ (1+I_k_)^L^_k_)/((1+I_k_)^(L^_k_^-1)^)] * [(Q_k_*θ_k_) + ((Q_k_* θ_k_* δ)/(1+I_k_)^L^_k_))} ……………………………………………………………….2

Where C_ω (capital)_ = Total capital cost for child health

A_ω_ = Space utilized (sq. Feet) at different health facility level for child health

φ_ω_ = Cost of one unit square feet

I_k_ = Annual Interest rate

L_k_ = Average life

Q_k_ = Quantity

θ_k_ = Unit price

δ = Annual maintenance rate

ω = 1(District Hospital), 2(First Referral unit) etc.

k = k_1_, k_2_ .............k_n_ (equipments)

This method (equation 2) was used to obtain the annualized cost of the infrastructure. We used the Government of Haryana rate contract prices for equipments and other capital infrastructure non-consumables. For the buildings etc, we estimated the floor space of all rooms and other areas in the respective health care facility. Total cost of building for under-5 child health care activities was estimated by multiplying the total floor space area with the rental price of the land in the given area and the proportion of time which was spent for child health care in that room. For rooms which were used for providing services to children alone, entire cost was allocated to U-5 healthcare. For space which was jointly used for child health and services to other age group population, proportion of time spent on child health care was apportioned by the fraction of pediatric U-5 children to total patients in the given health facility.

***Medicine, Diagnostic and Overhead costs***

Overhead costs, cost of total medicine consumption and the number of diagnostic (laboratory and radiological) tests were also apportioned to child health by a factor of pediatric to total patients in the given health facility.

**Medicines**

^U5^C_drugs_ = Σ_ω_ (q_i (annual)_ * p_i (drugs)_* P^U5^) …………………………………………..3

Where ^U5^C_drugs_ = Total cost of drugs at different health facility levels for child health

q_i (annual)_ = Quantity of i^th^ drug consumed in a year

p_i (drugs)_ = Unit price of i^th^ drug

P^U5^ = Proportion of under -5 year patient load (out of total)

i = i_1_, i_2_..........i_n_ (drugs)

ω = 1(District Hospital), 2(First Referral unit) etc

**Diagnostic**

^U5^C_diag_. = Σ_ω_ (N_i (annual)_ * p_i (diag.)_ * P^U5^) …………………………………………4

Where ^U5^C_diag_. = Total cost of diagnostic tests at different health facility levels for child health

N_i (annual)_ = Number of diagnostic tests of i^th^ type in a month

p_i (diag.)_ = Unit price of i^th^ diagnostic test

P^U5^ = Proportion of under-5 year patient load (out of total)

i = i_1_, i_2_..........i_n_ (diagnostic tests)

ω = 1(District Hospital), 2(First Referral unit) etc

**Overheads**

^U5^C_(over)_ = Σ_iω_ (C_i (over)_ * P^U5^) ................................................................................5

Where ^U5^C_(over)_ = Total overhead costs at different health facility levels for child health

C_i (over)_ = different overhead costs at each facility level

P^U5^ = Proportion of under-5 year patient load (out of total)

i = i_1_, i_2_..........i_n_ (different overhead costs)

ω = 1(District Hospital), 2(First Referral unit) etc

***District Support***

The costs included under district support included costs for supervision and training at district level for implementation and monitoring of child health programs. The supervisory costs include those for the staff, infrastructure, equipments, travel and space for the staff involved in under-5 child health care. Staff time, space, equipments costs were analyzed in the same manner as described above. For travel, we estimated the annual utilization, in terms of kilometers travelled, of the vehicles for staff assigned to activities related to child health care. For staff involved in supervision of both child and adult health programs, we apportioned their travel for child health by a factor of the proportion of under-5 year old population (among total population). Thereafter we applied market rental price for similar vehicles in district Faridabad to arrive at the economic cost of undertaking supervisory visits during the past one year.

***Program Support***

Cost of additional staff (staff time, space, consumables and non-consumables, and travel) which was recruited for the IMNCI implementation and monitoring in the 9 Primary Health Centres (PHC) in district Faridabad was also included in the district support cost. This cost was believed to be essential for monitoring the implementation of IMNCI at district level.

Training costs included costs of space, training materials, equipments, and honorarium for participants and resource persons. Additionally, we also estimated the time spent by each of the training participant and resource person. Certain trainings conducted in district were common to staff from intervention and control area. The total cost of these trainings was apportioned to each area, in proportion of trainees from respective area. Certain trainees were posted in district administration and contributed towards both intervention and control area. Training costs of these participants were apportioned to intervention and control area in a ratio of overall under-5 child population of the two areas respectively. Attendance in trainings does account for time cost as these trainees would have been performing some service during that time, had there been no training. Hence we accounted for the opportunity cost of time spent in training by multiplying the time spent by participant and resource persons with their respective gross salaries. For IMNCI trainings conducted in intervention area, certain resource persons were hired on contractual basis. Time costs of such resource persons were not added as these were external to the health system and their time was being compensated through the payment of honorarium.

Certain trainings conducted for health personnel from both intervention and control areas were beneficial for both child and adult health. Cost of such trainings were apportioned towards under-5 years child health as either proportion of live births among a total of live births and total pregnancies (Skilled Birth Attendance training), or as proportion of under-5 years old population among total population (HMIS and ASHA trainings). We acknowledge that trainings such as IUCD (Intra-uterine contraceptive device) training, which were mainly confined to training health workers on spacing methods of contraception, do impact on child survival through its indirect effect as part of life cycle approach in reproductive health. However, we did not cost for such training as part of child health program.

^U5^C_DS_ = Σ_m_( ^U5^S_ωm (annual)_ * N_m_ * F * ^U5^P_m_) + Σ_ω_ (A_ω_ * φ_ω_ * ^U5^P_ω_) + Σ_ωk_{([(I_k_ (1+I_k_)^L^_k_)/((1+I_k_)^(L^_k_^-1)^)] * [(Q_k_*θ_k_) + ((Q_k_* θ_k_* δ)/(1+I_k_)^L^_k_))} ……………………………6

Where ^U5^C_DS_ = Total cost of district support for child health

S_ωm (annual)_ = Annual salary of district level staff

N_m_ = Number of employees

F = Fixed time equivalent

^U5^P_m_/ ^U5^P_ω_ = Proportion of time spent on under-5 child health (out of total time)

A_ω_ = Space utilized (sq. Feet) at different health facility level for child health

φ_ω_ = Cost of one unit square feet

I_k_ = Annual Interest rate

L_k_ = Average life

Q_k_ = Quantity of capital equipment

θ_k_ = Unit price

δ = Annual maintenance rate

ω = 1(District Hospital), 2(First Referral unit) etc.

m = m_1_, m_2_.........m_n_ (category of staff)

k = k_1_, k_2_ .............k_n_ (equipments)

District support also includes the cost of IEC (wall paintings, pamphlets, TV/ radio advertisements etc) material which was procured under both intervention and control scenarios.

***Treatment seeking behaviour and Out-of-pocket costs for child health care***

Out-of-pocket (OOP) costs borne by households for treatment of infants were derived by analyzing data from Bhandari et al (BMJ-2012) and Mazumder et al (BMJ-2014).

A total of 37741and 39846 pregnancies in the intervention and control area were documented respectively in the cluster-randomized trial and 33122 and 34511 pregnancies were tracked for outcome. The other pregnancies could not be followed up till outcome as study ended. Nearly 53.4% and 51.9% of all the live births were followed till 1year of age. The other infants did not complete 12 months of age when study ended. Each live-birth was followed on day 29, 6 months and 1 year to ascertain the health status, presence of any disease, treatment seeking behaviour and costs incurred out-of-pocket by the household for treatment in the neonatal period at 29 days and previous 14 days at 6 months and 12 months visits. We analyzed this data on 12367, 6121 and 4062 children at 29 days, 6 months and 1 year age to ascertain the OOP costs. The out-of pocket costs include money spent on medicines, diagnostics, consultation charges paid to doctor, bed-charges paid for admission, procedures, transportation to health facility, and loss of productivity on account of the parents’ absence from work. Loss of productivity was based on the monthly salary and the number of days spent away from work for child care.

Average per infant out-of-pocket costs estimates were obtained for each level of health facility in both public and private sector health facilities.

**Standardizing costs at District level with and without IMNCI**

In order to compute the total economic costs for a standard district, we estimated the per-unit cost of child health care at each of the following levels:

1. ***Per unit cost of under-5 child treatment at district hospital***

This included per unit cost of curative care for in-patient admissions (IPD) at the district level. Per-unit cost was estimated by dividing the total cost of care on capital infrastructure, staff, medicines, diagnostics, referral and overheads for under-5 child admissions with the number of admissions during the year 2010.

^U5^θ_DH_ = [^U5^C_(salary)_ + ^U5^C_(capital)_ + ^U5^C_drugs_ + ^U5^C_diag_. + ^U5^C_(over)_]/β ..................................7

Where ^U5^θ_DH_ = Per unit cost of under-5 treatment at district hospital

β = Number of under-5 admissions during the year 2010

Per unit cost for OPD consultation was assumed to be 15% of the per unit IPD costs (GBD/WHO-CHOICE).

1. ***Per unit cost of under-5 child treatment at First Referral Unit (FRU/CHC)***

This was estimated in a similar manner as per unit cost for district hospital.

1. ***Per unit cost of under-5 child treatment at health centre (PHC)***

We parameterized our excel model for costing with the per unit cost of child care treatment at health centre level estimates from the Global Burden of Disease study for health centre level in South East Asia region (Ref: GBD) and local studies (IJCM study).

1. ***Per child cost of delivering under-5 child health services at multi-purpose health worker (MPHW), anganwadi (village-level child care worker) and ASHA (accredited social health activist, a village-level community worker) level.***

Data obtained on resource consumption at primary care level along with utilization data was thus analyzed to obtain per unit cost of child health care. Since the primary care workers devote much of their time to preventive and promotive care which is not limited to the sick children, hence the per unit cost was estimated at the total population level i.e. cost per under-5 year old child.

1. ***Standardized cost per District, with and without IMNCI***

Data on morbidity profile and treatment seeking behaviour of intervention and control area were used from the study Mazumder et al (BMJ-2014). In our analysis, we used the population parameters from district Faridabad to standardize cost calculations. Following equation was used to derive the cost of care at a standardized district level:

a) Cost of district support in a standardized district = (^U5^θ_DS/_ ^U5^ρ) * ^U5^Z …………….8

Where ^U5^θ_DS_ = Per child cost of district support in area ‘A’

^U5^ρ = Under-5 population of area ‘A’

^U5^Z = Infant population of standardized district

b) ^U5^C_OPD_ = Σ (^U5^C_HS(OPD)_ + C_OOP(x)_ )*( ^U5^μ * I_avg_. * P_ST_ * H_avg_. * P_ST(OPD)_ * ^U5^P_visits(x)_ ) …..9

Where ^U5^C_OPD_ = Cost of OPD care for under-5 children in a standardized district

^U5^C_HS(OPD)_ = Health system cost of OPD care in facility ‘x’

C_OOP(x)_ = Out of pocket cost for care in facility ‘x’

^U5^μ = Infant morbidity rate in area ‘A’

I_avg_. = Average number of illness episodes per infant per year in area ‘A’

P_ST_ = Proportion of infant who seek treatment in area ‘A’

H_avg_. = Average number of health providers visit per infant in area ‘A’

P_ST(OPD)_ = Proportion of infant who sought care in outpatient clinic

^U5^P_visits(x)_ = Proportion of infant visits to facility ‘x’

c) ^U5^C_IPD_ = Σ (^U5^C_HS(IPD)_ + C_OOP(x)_ )*( ^U5^μ * I_avg_. * P_ST_ * H_avg_. * P_ST(IPD)_ * ^U5^P_visits(x)_ ) ….10

Where ^U5^C_IPD_ = Cost of IPD care for under-5 children in a standardized district

^U5^C_HS(IPD)_ = Health system cost of IPD care in facility ‘x’

C_OOP(x)_ = Out of pocket cost for care in facility ‘x’

^U5^μ = Infant morbidity rate in area ‘A’

I_avg_. = Average number of illness episodes per infant per year in area ‘A’

P_ST_ = Proportion of infant who seek treatment in area ‘A’

H_avg_. = Average number of health providers visit per infant in area ‘A’

P_ST(IPD)_ = Proportion of infant who sought care in Inpatient clinic

^U5^P_visits(x)_ = Proportion of infant child visits to facility ‘x’

d) ^U5^C_immz_ = Σ (^U5^ρ_elg(i)_ * V_i(a)_ * C_i_ * W_i_ ) .................................................................11

Where ^U5^C_immz_ = Cost of under-5 immunization in a standardized district

^U5^ρ_elg(i)_ = Eligible population of children in standard district for vaccine ‘i’

V_i(a)_ = Vaccine coverage for vaccine ‘i’ in area ‘A’

C_i_ = Cost per unit dose of vaccine ‘i’

W_i_ = Wastage for vaccine ‘i’

Cost of child health care in a standardized district with IMNCI (C-IMNCI) = (^U5^θ_DS/_ ^U5^ρ) * ^U5^Z + ^U5^C_OPD_ + ^U5^C_IPD_ + ^U5^C_immz_) ……………………………………………12

Cost of child health care in a standardized district without IMNCI (C-Non IMNCI) = (D+ OPD + IPD + V) ……………………………………………………………………13

Incremental cost of implementing IMNCI in a standardized district = (C-IMNCI) – (C-Non IMNCI)

**Limitations**

In the past studies, the RCTs to determine impact and cost effectiveness of IMNCI have been done in different districts. Hence the hospital costs were different for both intervention and control districts. In our case, the RCT was done by randomizing the PHCs of a given district. The IMNCI model implemented in district Faridabad had a strong facility up-gradation at district level. However, since the benefits of district health system strengthening (i.e. development of Special Care Newborn Unit (SCNU) and Neonatal Stabilization Unit (NSU) at First Referral Unit (FRU) were reaped by both intervention and control area children and by similar logic, the costs of this additional upgradation was also shared between the two areas, based on usage. However since the cost of DH services were standardized based on usage, effect of IMNCI is implicitly included.

Secondly, cost data on joint trainings which were common to intervention and control areas was obtained for a period of 10 months. This was then extrapolated for the entire year to obtain costs of training over a 12 month period. Thirdly, since we costed for health system at one facility each, we could not make adjustments for capacity utilization. The bed occupancy of the facilities was 90% for district hospital and 70% for first referral unit. Capacity utilization for diagnostic services was nearly 75% in both facilities. Hence our cost estimates may not be generalized for very low utilization facilities. Fourth, we estimate per-unit cost of admission rather than bed-day cost of admission. Using the former for estimation of overall costs is inferior in the fact that it does not allow us an opportunity to model for a decline in the number of days of stay for an average patient in intervention and control area. Assuming that the IMNCI training leads to earlier referral by frontline workers, and improved care at health facility level, it can be safely assumed that there will be a reduction in the length of stay of children in intervention area. Since we did not have estimates of length of stay from the IMNCI impact assessment RCT study, we did not model this effect in the base analysis. Lastly, for standardizing the cost at district level, we assumed the unit cost of providing health care services to infants to be same as unit cost of U-5 child health care. We acknowledge that for any illness complication in the infancy age group the health care needed would be more critical than required for the U-5 child age group. Hence healthcare needed for infancy age group would be more time and resource intensive, increasing the average time spent for treating an infant as compared to U-5 child age group. Moreover, infant mortality accounts for more than 75% of the U-5 child mortality. Despite being aware of these facts, it was methodically very difficult to segregate the program costs of infants and U-5 child age groups. Also the package of services for 0-5 year child under IMNCI program are delivered utilizing the pooled resources without any differentiation.

**Valuation of Infant Health Benefits from IMNCI Program**

**IMNCI Impact Model**

We modeled the effects of IMNCI program on infant survival through reduction in morbidity and mortality following treatment. Years of life lost as a result of premature death in infancy (Years of Life Lost, YLL) and years of life lived in disability (YLD) as a result of disease or disability arising due to disease. Together the estimates for YLL and YLD were added to compute the disability adjusted life years (DALY) in both intervention and control areas. In order to estimate the incremental effect of implementing IMNCI program, we estimated the ‘DALYs averted’ as a difference of the DALYs lost when implementing IMNCI program from the DALYs lost when implementing routine child health program. We projected all effects to a standardized district, which for our analysis is district Faridabad in Haryana state, India.

As secondary endpoints, we also estimated the reduction in childhood illnesses and gain in life years as a result of reduction in premature childhood mortality. We present both undiscounted results, and those discounted at 3% rate.

**DALY Estimation**

We used the standard method for estimation of DALY lost as a result of infant illnesses. The following formula was used for estimation of YLL as a result of premature mortality:

$YLL=\frac{KCe^{ra}}{(r+\beta)^{2}}[e^{-(r+\beta)(L+a)}[-(r+\beta)(L+a)-1]-e^{-(r+\beta)a}[-(r+\beta)a-1]+\frac{1-K}{r}(1-e^{-rL})$ ………………..14

Where a= age at death in years; L = standard expectation of life in years at age a; r = discount rate; K = age-weighting modulation factor; C = age-weighting adjustment constant; β = constant from age weighting function

**Table A: Years of Life Lost Estimation Parameters**

| **Parameters** | **Infant Deaths** |
| --- | --- |
| Age weighting modulation factor (K) | 1 |
| Age weighting constant (β) | 0.166 |
| Discount rate (r) | 0.03 |
| Age at death (a) | 0.068 |
| Constant from the age weighting function (C) | 0.04 |
| Standard expectation of life at age (L) | 67 |
| E | 2.72 |
| YLL per infection averted | 32.14 |

*Source: The Global Burden of Disease concept*

Similarly the years of life lost as a result of disease and disability were calculated using the following formula:

$YLL=D\{\frac{KCe^{ra}}{(r+\beta)^{2}}[e^{-(r+\beta)(L+a)}[-(r+\beta)(L+a)-1]-e^{-(r+\beta)a}[-(r+\beta)a-1]]+\frac{1-K}{r}(1-e^{-rL})\}$ ………………15

Where a= age at death in years; D = disability weight; L = standard expectation of life in years at age a; r = discount rate; K = age-weighting modulation factor; C = age-weighting adjustment constant; β = constant from age weighting function

We used the commonly recommended discount rate of 3%, i.e. all future effects in terms of life years gained, illnesses averted and DALY averted were discounted at 3% each year (WHO Cost Effectiveness Analysis book). We applied age-weighting in calculation of DALY.

In order to estimate YLD as a result of morbidity, most common illnesses were identified in the infant age group, namely – diarrhoea and pneumonia and; severe illness/danger signs for neonatal age group. As diarrhoea, pneumonia and severe illness (neonatal) accounts for more than 90% of the overall illness burden among infants, therefore, no other illness was taken into consideration. YLD for each of illness was calculated. Moreover, all these illnesses in infancy age group which account for 90% of the infants’ illness burden, had a negligible contribution as YLDs in DALYs calculation as compared to YLLs, hence reflecting it to be an less important measure.

**Table B: YLD Estimations for Infant Morbidities**

| **Parameters** | **Infant age group** | | **Neonatal age group** |
| --- | --- | --- | --- |
|  | **Pneumonia** | **Diarrhoea** | **severe illness/danger signs** |
| Disability weight | 0.279 | 0.105 | 0.615 |
| Age weighting modulation factor | 1 | 1 | 1 |
| Age weighting constant | 0.166 | 0.166 | 0.166 |
| Discount rate | 0.03 | 0.03 | 0.03 |
| Age of onset of the disability | 0.16 | 0.16 | 0.16 |
| Constant from the age weighting function | 0.04 | 0.04 | 0.04 |
| Duration of disability | 0.022 | 0.022 | 0.022 |
| E | 2.72 | 2.72 | 2.72 |
| YLD per disability | 0.000185 | 0.0000695 | 0.000408 |

*Source: The Global Burden of Disease concept*

Overall DALY lost were computed as a sum of YLL (infants), YLD (infants diarrhoea), YLD (infant pneumonia), YLD (severe neonatal illness/danger signs).

Disease-wise disability rates from the Global burden of disease study were used to estimate the disability weights arising as a result of illness and long-term disability.

We calculated mean age of onset of infant death based on the assumption that 60% infant deaths occur in neonatal period, 60% of neonatal deaths occur in early-neonatal period, mean age of death in early neonatal period is 3 days, mean age of death in late-neonatal period is 20 days and mean age of post-neonatal infant death is 6 months. With these assumptions, we found that the mean age of infant death is 26 days.

**Table C**: IMNCI Impact Parameters on infant and child mortality, morbidity and disability

| Year | Infant Mortality Rate (Per 1000) | | Infant Morbidity Rate (%) | | | | Neonatal Morbidity Rate (%) | | Standard Population | Birth Rate (Per 1000) |
| --- | --- | --- | --- | --- | --- | --- | --- | --- | --- | --- |
|  |  |  | Pneumonia | | Diarrhoea | | Severe illness/Danger signs | |  |  |
|  | Intervention | Control | Intervention | Control | Intervention | Control | Intervention | Control |  |  |
| 2007 | 42.1 | 42.1 | 12.3 | 12.3 | 28.4 | 28.4 | 20.6 | 20.6 | 28171 | 23.7 |
| 2008 | 37.2 | 40.3 | 10.5 | 12.3 | 23.9 | 28.4 | 18.7 | 20.6 | 28628 | 23.3 |
| 2009 | 32.9 | 38.7 | 9.0 | 12.3 | 20.2 | 28.4 | 16.9 | 20.6 | 29093 | 23 |
| 2010 | 31.5 | 37.1 | 9.0 | 12.3 | 20.2 | 28.4 | 16.9 | 20.6 | 29565 | 22.6 |
| 2011 | 30.2 | 35.5 | 9.0 | 12.3 | 20.2 | 28.4 | 16.9 | 20.6 | 30044 | 22.3 |
| 2012 | 28.9 | 34 | 9.0 | 12.3 | 20.2 | 28.4 | 16.9 | 20.6 | 30532 | 22 |
| 2013 | 27.7 | 32.6 | 9.0 | 12.3 | 20.2 | 28.4 | 16.9 | 20.6 | 31027 | 21.6 |
| 2014 | 26.6 | 31.3 | 9.0 | 12.3 | 20.2 | 28.4 | 16.9 | 20.6 | 31531 | 21.3 |
| 2015 | 25.5 | 30 | 9.0 | 12.3 | 20.2 | 28.4 | 16.9 | 20.6 | 32042 | 21 |
| 2016 | 24.4 | 28.7 | 9.0 | 12.3 | 20.2 | 28.4 | 16.9 | 20.6 | 32562 | 20.7 |
| 2017 | 23.4 | 27.5 | 9.0 | 12.3 | 20.2 | 28.4 | 16.9 | 20.6 | 33090 | 20.4 |
| 2018 | 22.4 | 26.4 | 9.0 | 12.3 | 20.2 | 28.4 | 16.9 | 20.6 | 33627 | 20.1 |
| 2019 | 21.5 | 25.3 | 9.0 | 12.3 | 20.2 | 28.4 | 16.9 | 20.6 | 34173 | 19.8 |
| 2020 | 20.6 | 24.2 | 9.0 | 12.3 | 20.2 | 28.4 | 16.9 | 20.6 | 34727 | 19.5 |
| 2021 | 19.7 | 23.2 | 9.0 | 12.3 | 20.2 | 28.4 | 16.9 | 20.6 | 35291 | 19.2 |
| 2022 | 18.9 | 22.2 | 9.0 | 12.3 | 20.2 | 28.4 | 16.9 | 20.6 | 35863 | 18.9 |

In control area, reduction in IMR was estimated using the IMR estimates given by the three different rounds of National Family Health Survey (NFHS). These 3 rounds provide IMR estimates for the year 1992-93, 1998-99 and 2005-06. Logarithmic estimations were used to interpolate the IMR from the period 1992 to 2006 based on the rate of reduction observed in the 3 different rounds of NFHS. Same rate of reduction in IMR was applied to extrapolate the data from the period 2007 to 2022. For intervention area, reduction in IMR was applied using the findings from the India IMNCI effectiveness trial study, over and above the yearly IMR estimates in the control area (Bhandari et al, 2012, BMJ).

In control setting, we assumed no decline in infant (pneumonia and diarrhoea) and neonatal (severe illness/danger signs) morbidity rates from 2007 to 2022. For intervention arm, decline in infant and neonatal mortality was estimated only from 2007 to 2009 using the findings from India IMNCI effectiveness trial study (Mazumder et al, 2014, BMJ). For future years beyond the trial period i.e. 2009 to 2022, no decline in morbidity period was assumed but sustained levels achieved as a result of IMNCI.

We used the estimates of birth rate given by third round of District Level Household Survey (DLHS-III) for the base year i.e. 2007, in our study. An average relative decline of 1.5% in the birth rate was estimated using the Total Fertility rate (TFR) trends available in NFHS-III (Haryana rural). This rate of reduction was applied to extrapolate the birth rate from 2007 to 2022. Similarly, population from 2007 to 2022 was estimated using the growth rate available in Census 2001 data (Population Projections report, Census of India-2001).

**Figure A: Cost Effectiveness Analysis Plane (Health System perspective)**

**CEA Plane (Undiscounted)**


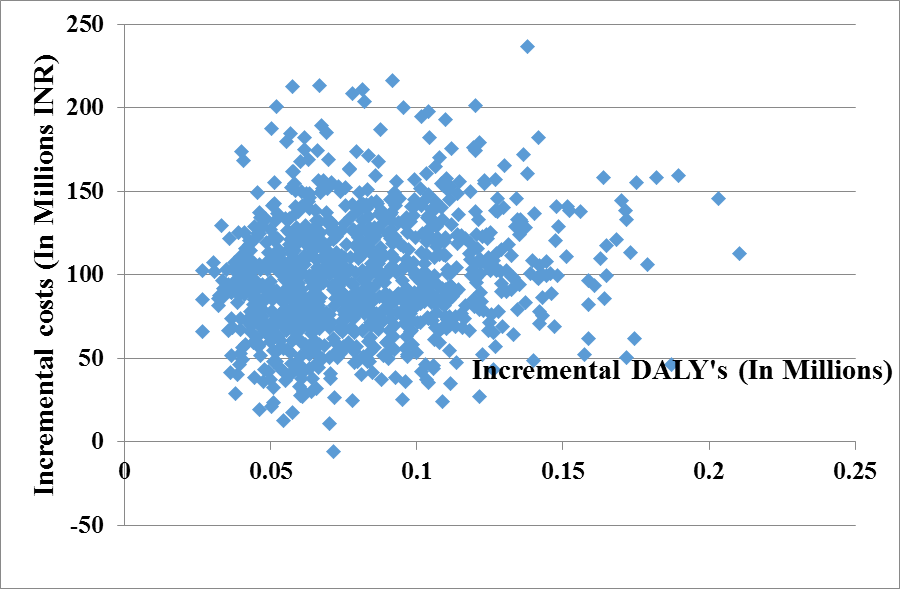

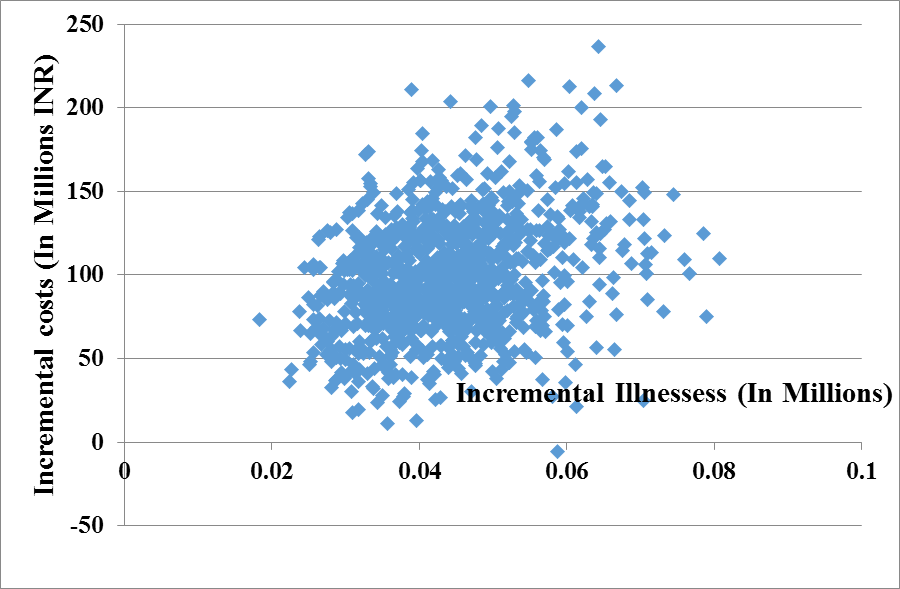


**CEA Plane (Discounted)**


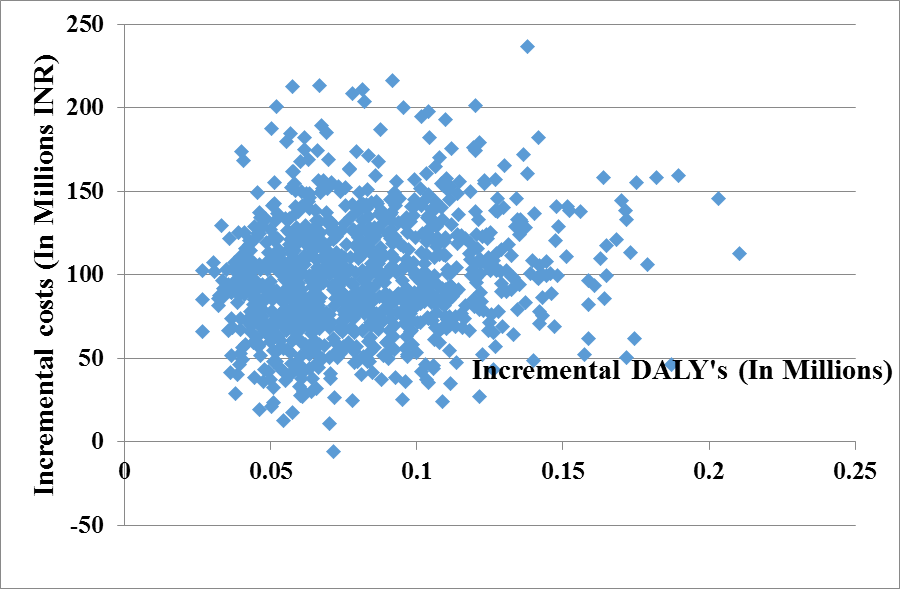

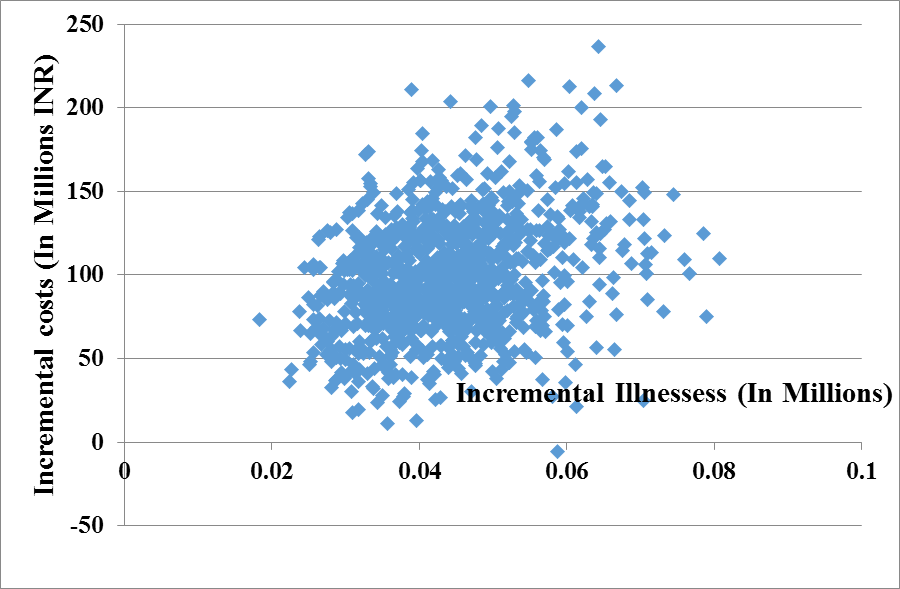


**Figure B: Cost Effectiveness Analysis Plane (Societal perspective)**

**CEA Plane (Undiscounted) CEA Plane (Discounted)**


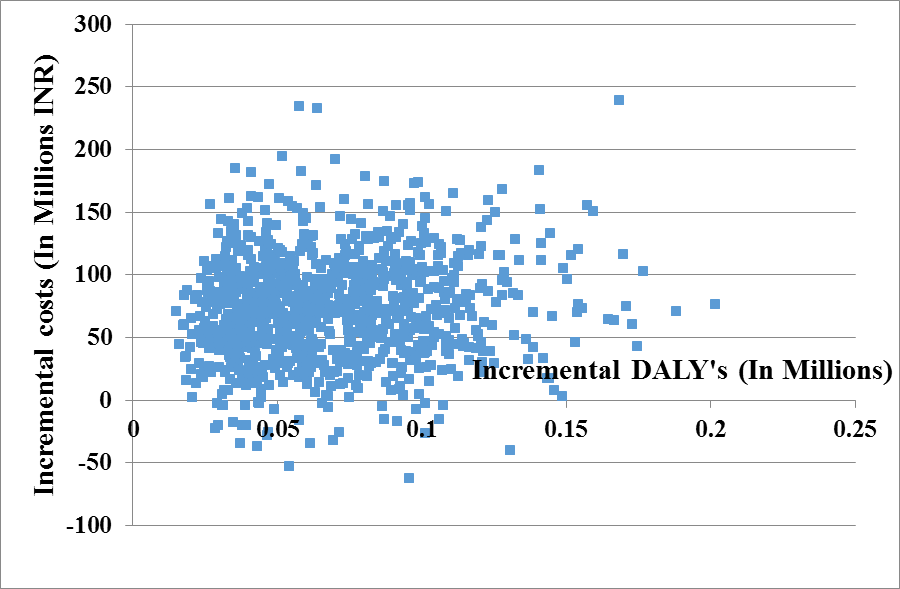

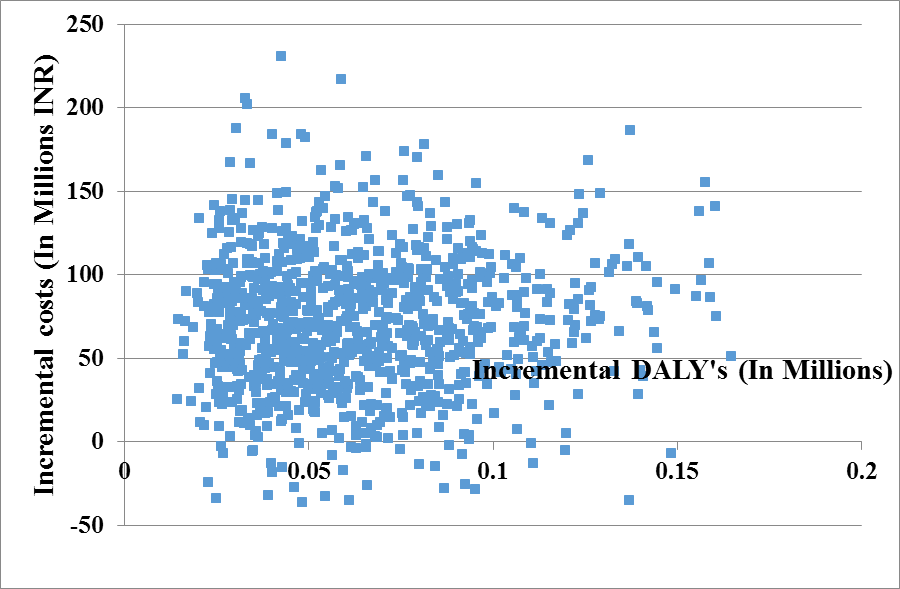

Supplement: S1 Material — (DOC) [file pone.0145043.s002.doc]
